# Reply to Foxon, F.; Shiffman, S. Comment on “Wang et al. Consumption of JUUL vs. Other E-Cigarette Brands among U.S. E-Cigarette Users: Evidence from Wave 5 of the PATH Study. *Int. J. Environ. Res. Public Health* 2022, *19*, 10837”

**DOI:** 10.3390/ijerph20186717

**Published:** 2023-09-06

**Authors:** Yu Wang, Zongshuan Duan, Scott R. Weaver, Lucy Popova, Claire A. Spears, David L. Ashley, Terry F. Pechacek, Michael P. Eriksen, Jidong Huang

**Affiliations:** 1 School of Public Health, Georgia State University, Atlanta, GA 30302, USA; ywang145@student.gsu.edu (Y.W.); srweaver@gsu.edu (S.R.W.); lpopova1@gsu.edu (L.P.); cspears@gsu.edu (C.A.S.); dashley4@gsu.edu (D.L.A.); tpechacek@gsu.edu (T.F.P.); meriksen@gsu.edu (M.P.E.); 2 Milken Institute School of Public Health, George Washington University, Washington, DC 20052, USA; zduan3@gwu.edu

In this reply, we respond to the comments by Foxon and Shiffman [1] regarding our study (Wang et al., ‘Consumption of JUUL vs. Other E-Cigarette Brands among U.S. E-Cigarette Users: Evidence from Wave 5 of the PATH Study [2]’). The primary criticism of Foxon and Shiffman was that we did not use replicate weights in our analysis of the PATH study data, and, had we used replicate weights instead of sampling weights, some of the study results would have become statistically insignificant.

Although we appreciate the comments raised by Foxon and Shiffman, the choice to use sampling weights was determined by our main research question, which was to provide nationally representative estimates of e-cigarette use by age groups (youth, young adults 18–24 years old, and adults aged 25 years and above) in the U.S. Using sampling weights was justified because they adjust the study sample to be nationally representative, and this was actually acknowledged by Foxon and Shiffman as “handled appropriately” in their comment. In fact, our estimates of JUUL use remain the same regardless of whether sampling weights or replicate weights were used, and our main conclusion of this study, that ‘JUUL consumption was disproportionally higher among youth and young adults in the U.S. in 2019,’ was not affected by using the sampling weights vs. the replicate weights.

That said, using replicate weights will take into account the PATH study’s complex, four-stage, stratified probability sample design, and could affect the variance estimations and the size of the estimated standard errors in multivariate regression analysis in Table 1 and Table 2 of our study, which in turn could potentially affect the statistical significance of the relationships reported in these two tables.

To address this criticism, we repeated the analyses in Table 1 and Table 2 using the replicate weights, following the User Guide of the PATH study [3] and using the balanced repeated replication (BRR) with a Fay coefficient of 0.3 to align with the methods employed in generating those weights. We also performed a re-analysis that generated the data for Figure 1 and Figure 2, as well as Figure S1 and Table S1. The updated figures and tables are listed below so that readers can examine the differences between the results of our original study, which were based on sampling weights, and the results presented in this Reply, which were based on replicate weights.

A careful review of these two sets of results revealed that our point estimates were not affected by using replicate weights vs. sampling weights. As such, no changes are reported in Figure 1. The point estimates in Figure 2 also stay the same, with only changes in the estimated standard errors. Similarly, all point estimates in Table 1 and Table 2 stayed the same, with only changes in the estimated standard errors and 95% confidence intervals (CIs). All statistically significant relationships in Table 1 and Table 2 remained significant when using replicate weights, except for three, which became statistically insignificant once the replicate weights were used. These three changes were marked with asterisks in the revised Table 1 and Table 2.

In summary, the primary results and conclusion of our study, that ‘JUUL consumption was disproportionally higher among youth and young adults in the U.S. in 2019′ were not affected by using replicate weights vs. sampling weights.

Below, we provided a detailed description of the differences, if any, between the results using sampling weights vs. replicate weights.

In Figure 1, no changes were found between the point estimates using sampling weights vs. replicate weights. As shown in Figure S1, among participants who used e-cigarettes in the past 30 days and knew the brand name, 65.2% of youth, 60.7% of young adults, and 25.6% of adults usually/last used JUUL. Among participants who usually/last used JUUL in the past 30 days, 13.4% were youth, 50.7% were young adults, and 35.9% were adults. Among participants who reported using other brands, 5.0% were youth, 22.8% were young adults, and 72.3% were adults. Of all past 30-day JUUL consumption measured in puffs, 15.4% was consumed by youth, 55.5% by young adults, and 29.1% by adults. By contrast, for the consumption of other e-cigarette brands, 4.2%, 28.9%, and 66.9% were consumed by youth, young adults, and adults, respectively (Figure 1).

In Figure 2, although the point estimates were not affected, the 95% CIs for the estimations of percentages of participants who used JUUL and other e-cigarette brands by use frequency and age group changed slightly due to the use of replicate weights (Figure 2). However, these changes do not alter the original conclusions of this study. The frequency of the distribution of use among youth was similar between JUUL and other e-cigarette brands. Adults who used other e-cigarette brands were less likely to use e-cigarettes rarely and more likely to use e-cigarettes daily, compared with adults who reported using JUUL.

In Table 1, all point estimates stayed the same. However, one change in statistical significance was identified when using replicate weights. In the original analysis, it was found that youth who reported current cigarette smoking were less likely to use JUUL (aOR = 0.55, 95% CI: 0.30–0.99). However, after applying the replicate weights, this association became statistically insignificant (aOR = 0.55, 95% CI: 0.27–1.12) (Table 1). All other statistically significant relationships remain the same with the significance level set at 0.05.

In Table 2, similarly, all point estimates stayed the same. However, two changes in statistical significance were identified when using the replicate weights. In the original analysis, it was found that youth who reported using JUUL were more likely to use e-cigarettes within 30 min after waking (aOR = 2.30, 95% CI: 1.12–4.75); and non-Hispanic other adults aged 25 years and above were less likely to use e-cigarettes within 30 min after waking (aOR = 0.48, 95% CI: 0.24–0.97). However, after applying the replicate weights, these associations became statistically insignificant (aOR = 2.30, 95% CI: 0.98–5.42; and aOR = 0.48, 95% CI: 0.23–1.04, respectively) (Table 2). All other statistically significant relationships remain the same with the significance level set at 0.05. Additional updated results of the descriptive statistics for the study sample are presented in Table S1.

In summary, we appreciate the comments raised by Foxon and Shiffman regarding the use of replicate weights vs. sampling weights in the analysis of the PATH study data. The analysis we conducted in response to Foxon and Shiffman’s comments demonstrated that the key conclusions of our study were not impacted by the use of replicate weights, and the choice of which weight to use depends on the key research questions in focus.

## Figures and Tables

**Figure 1:**
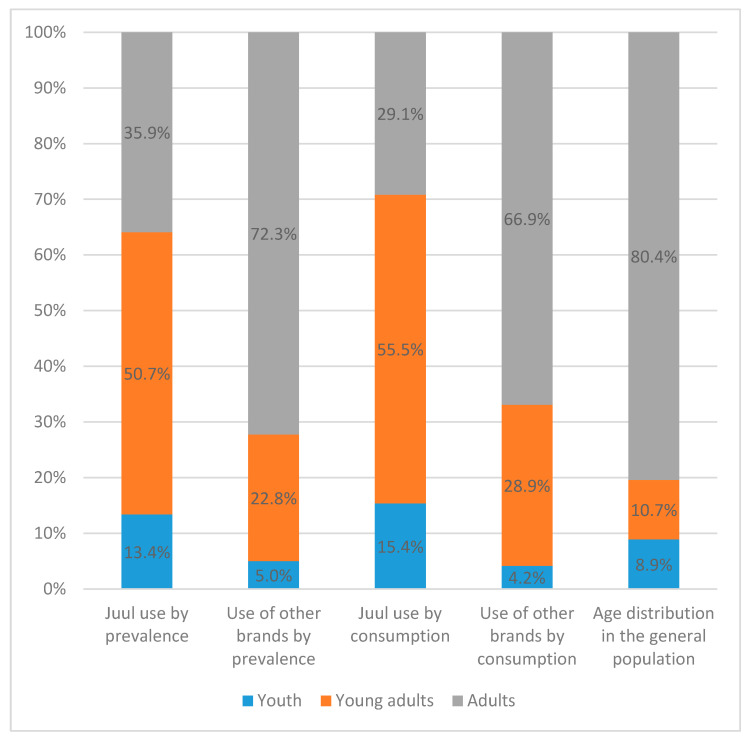
Distribution of JUUL use vs. use of other e-cigarette brands among U.S. youth, young adult, and adult e-cigarette users, compared with the proportion of population size.

**Figure 2:**
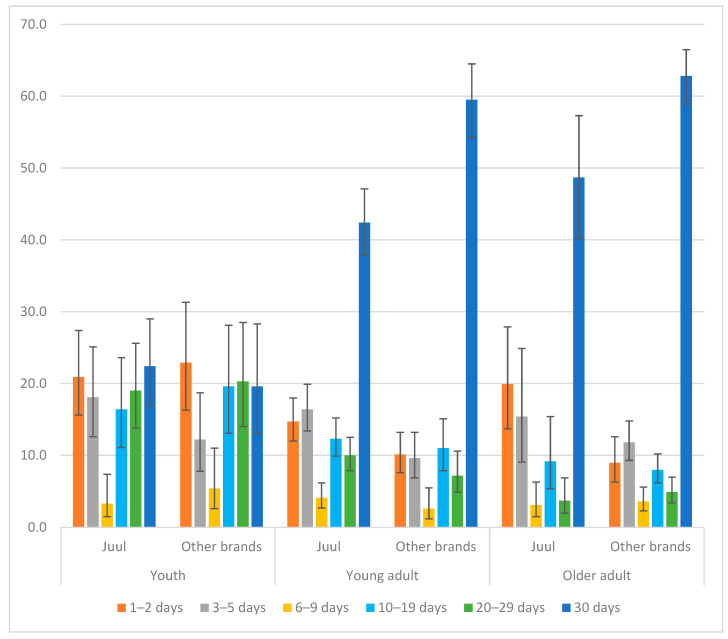
Percentages of JUUL users vs. users of other e-cigarette brands by use frequency and age group.

**Table 1:** Associations between JUUL use and e-cigarette use frequency, socio-demographic characteristics, and other tobacco use among U.S. youth, young adult, and adult e-cigarette users.

Individual Characteristics	Youth		Young Adults		Adults	
	OR	95% CI	OR	95% CI	OR	95% CI
E-cigarette use frequency	1.01	0.98–1.03	0.97	0.96–0.98	0.97	0.96–0.99
Sex						
Male	0.63	0.38–1.05	0.99	0.75–1.31	1.39	0.88–2.18
Female	Ref.		Ref.		Ref.	
Race/ethnicity						
Non-Hispanic White	Ref.		Ref.		Ref.	
Non-Hispanic Black	0.55	0.12–2.59	1.29	0.73–2.28	0.38	0.16–0.88
Hispanic	0.67	0.39–1.16	0.64	0.44–0.95	0.76	0.36–1.58
Non-Hispanic other	1.18	0.52–2.65	0.77	0.44–1.35	0.31	0.14–0.68
Education/Parental education						
Less than high school	0.58	0.30–1.12	0.29	0.15–0.53	0.28	0.14–0.59
High school graduate	0.49	0.18–1.36	0.34	0.19–0.61	0.42	0.24–0.76
Some college or associate degree	0.63	0.35–1.13	0.54	0.31–0.93	0.48	0.29–0.79
Bachelor’s degree or above	Ref.		Ref.		Ref.	
Cigarette smoking						
Yes	0.55	0.27–1.12 *	0.85	0.59–1.22	1.16	0.78–1.73
No	Ref.		Ref.		Ref.	
Other tobacco use						
Yes	0.54	0.24–1.20	0.45	0.33–0.62	0.75	0.41–1.38
No	Ref.		Ref.		Ref.	

* Inconsistency in statistical significance from the original results.

**Table 2:** Association between e-cigarette dependency (measured as using e-cigarettes within 30 min after waking) and JUUL use among U.S. youth, young adults, and adults.

Individual Characteristics	Youth		Young Adults		Adults	
	OR	95% CI	OR	95% CI	OR	95% CI
JUUL user						
Yes	2.30	0.98–5.42 *	1.17	0.84–1.63	0.57	0.36–0.89
No	Ref.		Ref.		Ref.	
E-cigarette use frequency	1.14	1.10–1.18	1.10	1.08–1.13	1.07	1.05–1.09
Sex						
Male	1.18	0.54–2.57	1.29	0.85–1.98	0.72	0.50–1.02
Female	Ref.		Ref.		Ref.	
Race/ethnicity						
Non-Hispanic White	Ref.		Ref.		Ref.	
Non-Hispanic Black	1.18	0.20–7.16	0.47	0.20–1.11	0.85	0.47–1.55
Hispanic	1.09	0.42–2.83	0.77	0.44–1.36	0.48	0.24–0.98
Non-Hispanic other	0.99	0.30–3.24	0.67	0.38–1.20	0.48	0.23–1.04 *
Education/Parental education						
Less than high school	0.96	0.24–3.87	1.25	0.61–2.55	1.65	0.82–3.30
High school graduate	1.69	0.74–3.86	1.30	0.64–2.62	1.17	0.61–2.27
Some college or associate degree	0.80	0.36–1.79	0.97	0.52–1.81	1.21	0.75–1.96
Bachelor’s degree or above	Ref.		Ref.		Ref.	
Cigarette smoking						
Yes	2.02	0.89–4.56	1.12	0.72–1.74	0.70	0.47–1.05
No	Ref.		Ref.		Ref.	
Other tobacco use						
Yes	1.90	0.46–7.80	1.56	1.06–2.30	1.23	0.80–1.88
No	Ref.		Ref.		Ref.	

* Inconsistency in statistical significance from the original results.

## Supplementary Materials

The following supporting information can be downloaded at: https://www.mdpi.com/article/10.3390/ijerph20186717/s1, Figure S1: Proportion of respondents who knew the e-cigarette brand names they frequently/last used among U.S. youth, young adult, and adult past 30-day e-cigarette users in 2019 (PATH Wave 5).; Table S1: Descriptive statistics of past 30-day e-cigarette users who know the brand names they usually/last used among U.S. youth, young adults, and adults.
