# Supplementary material for: Reply to Foxon, F.; Shiffman, S. Comment on “Wang et al. Consumption of JUUL vs. Other E-Cigarette Brands among U.S. E-Cigarette Users: Evidence from Wave 5 of the PATH Study. Int. J. Environ. Res. Public Health 2022, 19, 10837”
Source: Int J Environ Res Public Health. 2023 Sep 6;20(18):6717. doi: 10.3390/ijerph20186717 (PMC10531448; doi:10.3390/ijerph20186717)
Supplement: Supplementary file 1 [file ijerph-20-06717-s001.zip › ijerph-2442503-supplementary.pdf]

International Journal of
Environmental Research and Public Health

MDPI

Figure S1. Proportion of respondents who knew the e-cigarette brand names they frequently/last used among U.S. youth, young adult, and adult past 30-day e-cigarette users in 2019 (PATH Wave 5).

1a. Youth (Unweighted frequency: 390 youth reported the brand names of e-cigarette they usually/last used, 243 reported JUUL.)

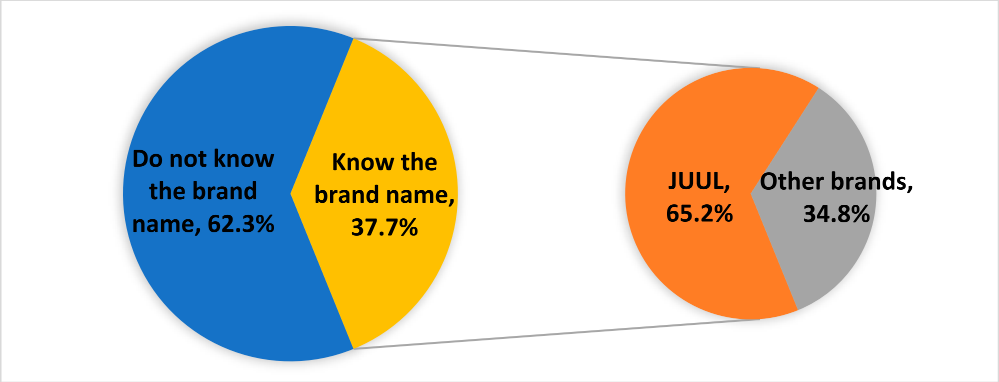

1b. Young adults (Unweighted frequency: 1,183 young adults reported the brand names of e-cigarette they usually/last used, 699 reported JUUL)

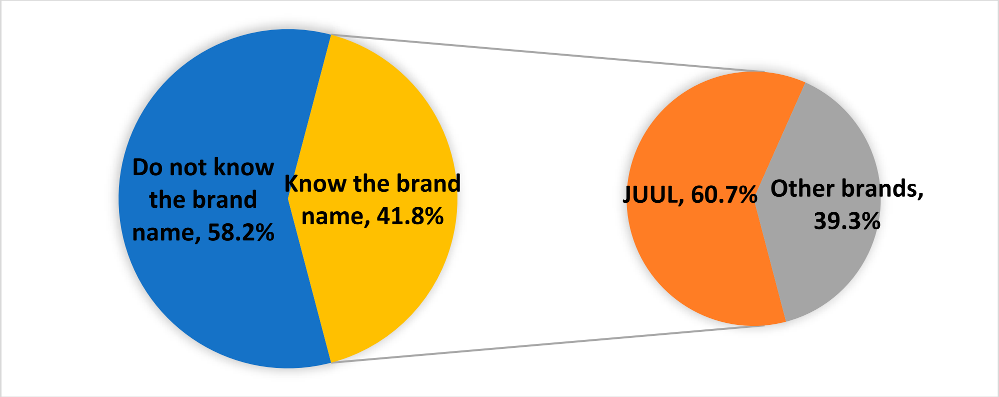

1c. Adults (Unweighted frequency: 996 adults reported the brand names of e-cigarette they usually/last used, 238 reported JUUL)

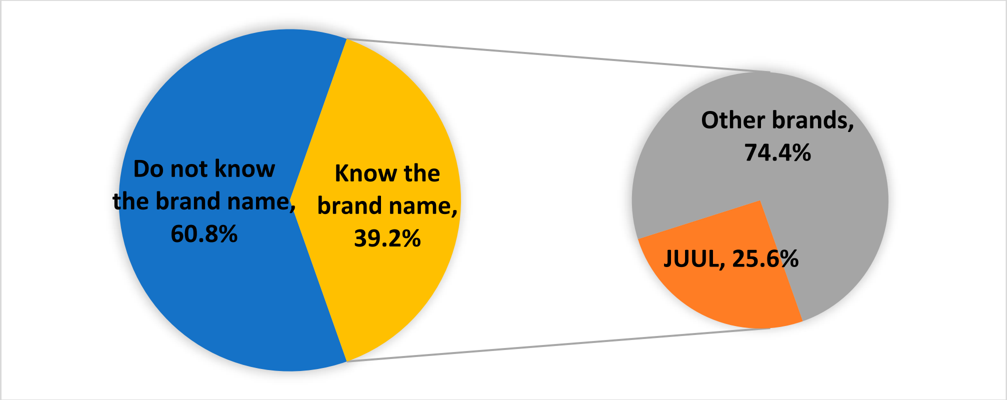

Table S1. Descriptive statistics of past 30-day e-cigarette users who know the brand names they usually/last used among U.S. youth, young adults, and adults.

	Youth		Young adults		Adults	
Individual characteristics	%	95% CI	%	95% CI	%	95% CI
Male	54.2	48.5–59.8	62.3	59.1–65.4	54.9	50.8–58.9
Female	45.8	40.2–51.5	37.7	34.6–40.9	45.1	41.1–49.2
Race/ethnicity						
Non-Hispanic White	69.5	63.5–74.9	75.0	72.3–77.6	76.0	72.8–78.9
Non-Hispanic Black	3.0	1.7–5.2	5.1	3.9–6.6	7.9	5.9–10.5
Hispanic	17.7	13.8–22.5	11.7	10.2–13.5	9.4	7.4–12.0
Non-Hispanic other	9.8	6.9–13.7	8.2	6.5–10.2	6.7	5.1–8.8
Education/Parental education						
Less than high school	11.3	7.8–16.1	13.7	11.4–16.3	14.4	12.0–17.3
High school graduate	15.9	12.2–20.4	28.3	25.3–31.4	26.9	23.5–30.6
Some college or associate degree	32.8	28.2–37.8	47.4	44.3–50.6	37.9	34.7–41.3
Bachelor's degree or above	40.1	34.6–45.7	10.7	8.2–13.7	20.7	17.8–24.1
Cigarette smoking						
Yes	23.7	19.2–28.8	29.8	27.1–32.7	52.8	49.0–56.6
No	76.3	71.2–80.8	70.2	67.3–72.9	47.2	43.4–51.0
Other tobacco use						
Yes	14.4	11.1–18.5	25.3	22.8–28.1	22.6	19.5–26.0
No	85.6	81.5–88.9	74.7	71.9–77.2	77.4	74.0–80.5
